# m6A-related lncRNA-based immune infiltration characteristic analysis and prognostic model for colonic adenocarcinoma

**DOI:** 10.1186/s41065-023-00267-y

**Published:** 2023-02-09

**Authors:** Hao-lun Wang, Zhuo-miao Ye, Zi-yun He, Lu Huang, Zhi-hui Liu

**Affiliations:** 1 Graduate School of Guangxi Medical University, Nanning, 530021 China; 2 Department of Oncology, Xiangya Hospital, Central South University, Changsha, Hunan 410008 China; 3 Day-Care Unit, Affiliated Cancer Hospital of Guangxi Medical University, Nanning, 530021 China

**Keywords:** Immune infiltration, Prognostic model, m^6^A-related long non-coding RNA, Colonic adenocarcinoma

## Abstract

**Background:**

Colonic adenocarcinoma (COAD) is a common gastrointestinal tract tumor, and its occurrence and progression are typically associated with genomic instability, tumor-suppressor gene and oncogene mutations, and tumor mutational load. N6-methyladenosine (m^6^A) modification of RNAs and long non-coding RNA (lncRNA) expression are important in tumorigenesis and progression. However, the regulatory roles of m^6^A‐associated lncRNAs in the tumor microenvironment, stratification of prognosis, and immunotherapy are unclear.

**Methods:**

We screened 43 prognostic lncRNAs linked to m^6^A and performed consistent molecular typing of COAD using consensus clustering. The single-sample Gene Set Enrichment Analysis and ESTIMATE algorithms were used to assess the immune characteristics of different subgroups. Covariation between methylation-related prognostic lncRNAs was eliminated by least absolute shrinkage and selection operator Cox regression. A nomogram was created and evaluated by combining the methylation-related prognostic lncRNA model with other clinical factors. The relationship between the prognostic model grouping and microsatellite instability, immunophenotype score, and tumor mutation burden was validated using R scripts. Finally, we used a linkage map to filter sensitive medicines to suppress the expression of high-risk genes.

Three m^6^A-associated lncRNA modes were identified in 446 COAD specimens with different clinical endpoints and biological statuses. Risk scores were constructed based on the m^6^A-associated lncRNA signature genes. Patients with lower risk scores showed superior immunotherapy responses and clinical benefits compared to those with higher risk scores. Lower risk scores were also correlated with higher immunophenotype scores, tumor mutation burden, and mutation rates in significantly mutated genes (e.g., *FAT4* and *MUC16*). Piperidolate, quinostatin, and mecamylamin were screened for their abilities to suppress the expression of high-risk genes in the model.

**Conclusions:**

Quantitative assessment of m^6^A-associated lncRNAs in single tumors can enhance the understanding of tumor microenvironment profiles. The prognostic model constructed using m^6^A-associated lncRNAs may facilitate prognosis and immunotherapy stratification of patients with COAD; finally, three drugs with potential therapeutic value were screened based on the model.

**Supplementary Information:**

The online version contains supplementary material available at 10.1186/s41065-023-00267-y.

## Background

Colorectal cancer (CRC) is a general term used to describe malignant tumors of colon epithelial origin, and Colonic adenocarcinoma (COAD) is the most common histological type of colon cancer. COAD is a common gastrointestinal tumor with the third and fourth highest incidence and mortality rates, respectively [1, 2]. More than 1 million patients are newly diagnosed with COAD each year, among which approximately 600,000–700,000 patients die from COAD [2, 3].

Its occurrence and development are typically related to genomic instability, mutations in tumor-suppressor genes and oncogenes, expression disorders, tumor mutation burden, and other factors. CRC,including COAD is highly heterogeneous not only at the genetic level but also at the molecular level [4]. This heterogeneity strongly influences the prognosis of patients and effectiveness of available immunotherapy. Understanding the molecular principles of COAD development and progress may lead to improvements in the diagnosis and treatment of this disease.

The tumor microenvironment (TME) refers to the environment containing tumor or cancer stem cells and molecules. It increases tumor cell stemness, promotes angiogenesis, mediates migration, reduces drug sensitivity, and inhibits the autoimmune defense system. A better understanding of the function and molecular biology of the TME can provide important insights into different tumors. Additionally, new cancer therapies have been developed that target cancer-promoting processes and molecules in the TME [5].

Immunotherapy stimulates the ability of the immune system to fight cancer cells; particularly, immunotherapy with PD-1/PD-L1 is among the most promising approaches for treating CRC. In patients with CRC characterized by mismatch repair deficiency (DMMR) or microsatellite instability (MSI) mutations, tumors often have a high mutation burden, abundant tumor-infiltrating lymphocytes, and upregulated PD-L1 expression in the TME. These factors lead to better prognosis following treatment with PD-1/PD-L1 [6, 7].

The m^6^A methylation modification in RNA, which occurs at the methyl adenosine N6 position, brings about a broad and abundant change in mRNA and non-coding RNA (ncRNA), dynamic regulating tumor genesis and development [8]. m^6^A is dynamically adjusted by specific methyltransferases (writers) and demethylases (erasers). Mutations and disorders in these enzymes are often related to occurrence, progression, metastasis, and recurrence of tumors [9].

M^6^A methylation often occurs in the poly(A) regions of long ncRNAs (lncRNAs). An imbalance in m^6^A modification may lead to abnormal expression of lncRNAs, which can regulate gene expression through transcriptional and histone modifications; increase chromosomal instability; and participate in cancer cell growth, metastasis, and drug resistance [9, 10] to regulate tumor progression. Screening for prognostic lncRNAs associated with m^6^A can reveal important indicators for evaluating tumor prognosis [11].

M^6^A modifications play integral roles in inflammation, innate immunity, and anti-tumor effects. Although some relevant studies conducted on a few m^6^A regulators [9, 12, 13], the roles of lncRNAs in TME regulation of COAD require further analysis. In addition, the occurrence and progression of highly heterogeneous COAD are associated with MSI, MMR, tumor mutational burden (TMB), or other biomarkers.

Therefore, evaluating a specific m^6^A-regulated lncRNA alone is insufficient. We aim to identify novel biomarkers to assess immune checkpoints and other relevant factors to comprehensively understand m^6^A-related lncRNA-mediated TME profiles and improve prognosis and immunotherapy stratification.

In this study, we evaluated genomic information from 514 CRC samples to comprehensively assess the modification patterns of m^6^A-associated lncRNAs and correlate the results with the infiltration characteristics of TME cells to systematically assess the relationships between m^6^A-associated lncRNAs and COAD prognosis, immune checkpoints, tumor immune infiltration, TMB, MSI, and immune scoring.m^6^A-related lncRNA-related risk models were established to stratify the prognosis of patients with COAD and facilitate treatment decisions. We also examined the regulatory mechanism between the TME and m^6^A, thus providing strategies for COAD immunotherapy.

## Results

### m^6^A regulator-associated prognostic lncRNAs in COAD

**Fig. 1:**
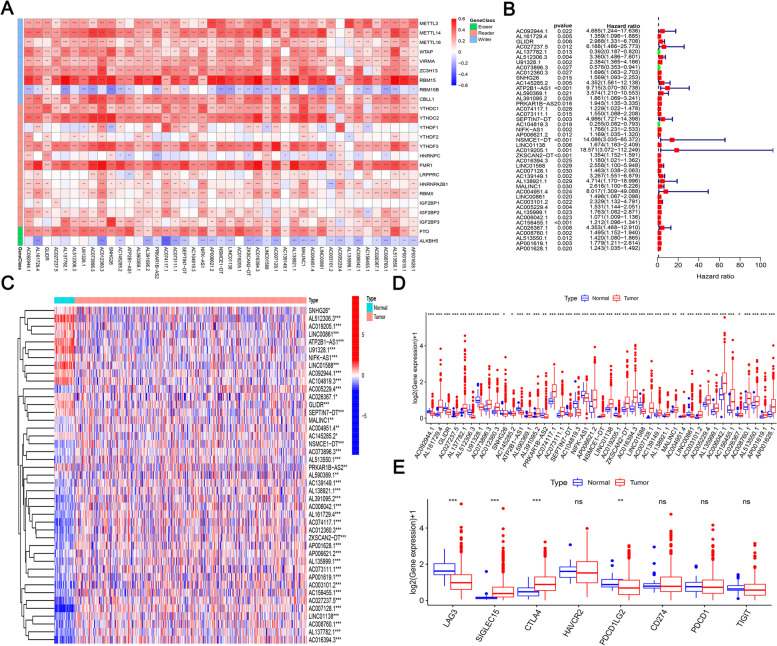
Correlation and survival risk of m^6^A-associated prognostic lncRNAs with m^6^A regulators and expression in COAD and adjacent tissues. **a** Heat map of correlations between m6A regulators and 43 m6A-associated lncRNAs in TCGA data using one-way Cox regression analysis. The m6A-related prognostic lncRNAs were positively correlated with m6A regulators except RBM15B and ALKBH5, both of which showed a negative relationship with m6A-related prognostic lncRNA. **b** Survival risk ratios of 43 m6A-associated prognostic lncRNAs related to m6A in TCGA database were significantly associated with overall survival (OS) in patients with COAD; all of these lncRNAs were negatively associated with OS except for AL137782.1, AC073896.3, and AC104819.3. **c** and **d** Heat map (**c**) and box plot (**d**) showed that m6A methylation-related prognostic lncRNAs expression in COAD tumors and adjacent normal tissues by using Cancer Genome Map (TCGA) data to systematically examine the expression of 43 m6A methylation-related prognostic lncRNAs in COAD and its adjacent normal tissues. These lncRNAs were significantly differentially expressed in COAD and normal tissues, showing overall upregulated expression. **e** Differential expression of immune checkpoints in COAD and paracancerous normal tissue species. Comparison of eight common immune checkpoints in COAD tumors and paracancerous tissues revealed inconsistent expression patterns. *P < 0.05, **P < 0.01, ***P < 0.001. COAD, colon adenocarcinoma; lncRNA, long non-coding RNA; m6A: N6-methyladenosine

First, to explore m^6^A regulator-associated lncRNAs, the expression matrices of 43 m6A regulators in The Cancer Genome Atlas (TCGA) database were extracted, and 14,086 lncRNAs were identified for subsequent analysis. We searched for m^6^A-associated lncRNAs in each dataset using Pearson’s correlation analysis. LncRNAs related to m^6^A were expected to show expression values related to at least one of the 24 regulatory factors of m^6^A (|Pearson R|> 0.4, *P* < 0.001). Our results showed that 1612 lncRNAs were significantly related to m^6^A methylation. LncRNAs that were significantly correlated with related genes were combined with prognostic information, and lncRNAs associated with m^6^A were selected using univariate Cox regression (*P* < 0.03). Finally, 43 lncRNAs related to m^6^A in TCGA database were significantly associated with overall survival (OS) in patients with COAD; all of these lncRNAs were negatively associated with OS except for *AL137782.1*, *AC073896.3*, and *AC104819.3* (Fig. 1b). In addition, all m6A regulators were positively correlated with m6A-associated prognostic lncRNAs, except for RBM15B and ALKBH5, which were negatively correlated with m6A-associated prognostic lncRNAs (Fig. 1a).

### Expression of lncRNA and immune checkpoints related to m^6^A methylation in COAD

To evaluate the biological functions of the lncRNAs associated with m^6^A methylation in the progression of COAD, we used Cancer Genome Map (TCGA) data to systematically examine the expression of 43 m6A methylation-related prognostic lncRNAs in COAD and its adjacent normal tissues. We obtained expression profiles from 473 tumor tissues and 41 paracancerous normal tissues and performed differential expression analysis of selected m^6^A regulator-associated prognostic lncRNAs. These lncRNAs were significantly differentially expressed in COAD and normal tissues, showing overall upregulated expression (Fig. 1c and d).

Except for *SNHG26*, the genes *AC145285.2*, *AC026367.1* (*P* < 0.05), *AC092944.1*, *GLIDR*, *AL512306.3*, *U91328.1*, *ATP2B1-AS1*, *PRKAR1B-AS2*, *SEPTIN7-DT*, *AC104819.3*, *NIFK-AS1*, *AC019205.1*, *LINC01588*, and *LINC00861* (*P* < 0.001) were abundant in normal tissues adjacent to the cancer, whereas the remaining genes were abundant in COAD tissues. In addition, we performed differential expression analysis of matchable lncRNAs by combining the GTEX database, and the expression of these M6A-associated prognostic lncRNAs differed significantly between normal and cancerous tissues (Figure S1). These results suggest that m^6^A methylation-associated prognostic lncRNAs play important roles in COAD progression. Comparison of eight common immune checkpoints in COAD tumors and paracancerous tissues revealed inconsistent expression patterns. The expression levels of *PDCD1LG2* (*P* < 0.01) and *LAG3* (*P* < 0.001) were higher in normal paracancerous tissues, whereas that of *SIGLEC15* with *CTLA4* (*P* < 0.001) was higher in COAD tissues. The remaining immune checkpoints showed no significant expression differences between tissues (Fig. 1e).

### Association of m^6^A methylation-related prognostic lncRNA consensus clustering with characteristics and survival of patients with COAD

**Fig. 2:**
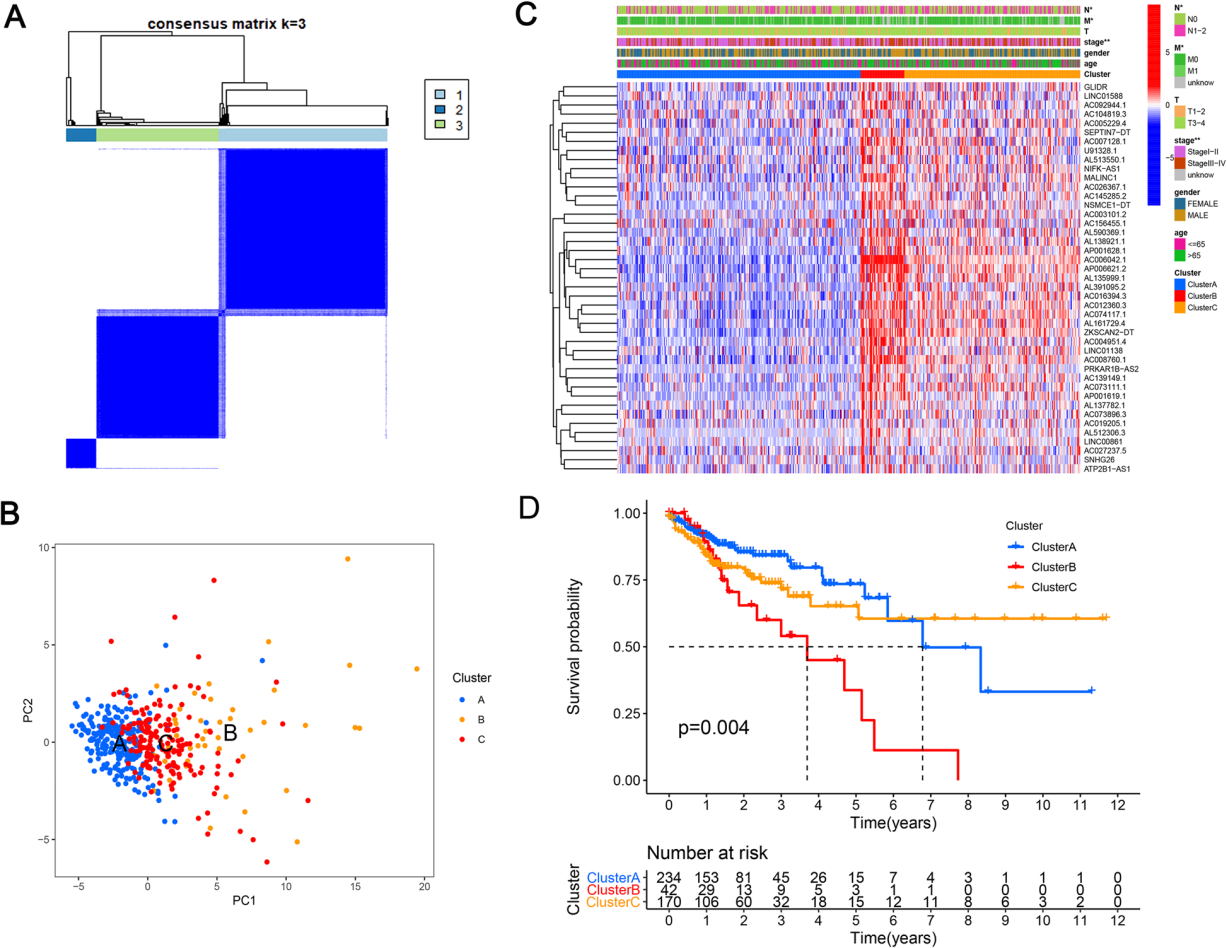
Clinical attributes and survival rates of COAD subtypes in clusters A–C. **a** Based on the similarity between the expression level of lncRNAs related to m6A regulators and proportion of fuzzy clustering measure, k = 3 was determined. **b** Principal component analysis was performed to further analyze the gene expression profile between the isomers,revealing differences among the three isomers. **c** Clinical correlation heatmap of clusters A–C and the clinical characteristics of the three subtypes were compared. The TNM staging ratio of cluster B was higher than that of gene clusters A and C (*P* < 0.01). Clusters A and C contained a lower proportion of patients with distant and lymph node metastases compared to that in cluster B (*P* < 0.05). **d** Kaplan–Meier curves for overall survival of patients with COAD in the 3 groups. The OS difference among lncRNA clusters of the three m6A methylation-related prognoses was significant. The OS rate in group B was significantly lower than that in the other two groups. *P < 0.05, **P < 0.01, ***P < 0.001. COAD, colon adenocarcinoma

Based on the similarity between the expression level of lncRNAs related to m^6^A regulators and proportion of clustering measure, k = 3 was determined, and the optimal clustering stability was k = 2–9 (Fig. 2a). Principal component analysis (PCA) was performed to further analyze the gene expression profile between the three subtypes, revealing differences among the three subtypes (Fig. 2b). According to the expression level of the m^6^A regulator, the 446 patients with COAD were divided into three subtypes: clusters A (*n* = 234), B (*n* = 42), and C (*n* = 170). Prognostic lncRNAs related to m^6^A methylation in group B were mostly significantly higher than those in the other two groups, particularly in group A (Fig. 2c).

The clinical characteristics of the three subtypes were compared (Fig. 2c). The proportion of patients in TNM stages 3 and 4 was higher in group B than in groups A and C (*p* < 0.01). Clusters A and C contained a lower proportion of patients with distant and lymph node metastases compared to that in cluster B (*P* < 0.05).

The log-rank *P*-value of the Kaplan–Meier curve was 0.004, indicating that the OS difference among lncRNA clusters of the three m^6^A methylation-related prognoses was significant (Fig. 2d). The OS rate in group B was significantly lower than that in the other two groups.

### Immuno-infiltrative infiltration characteristics and molecular characterization of consensus clustering typing of m6A methylated prognostic lncRNAs

**Fig. 3:**
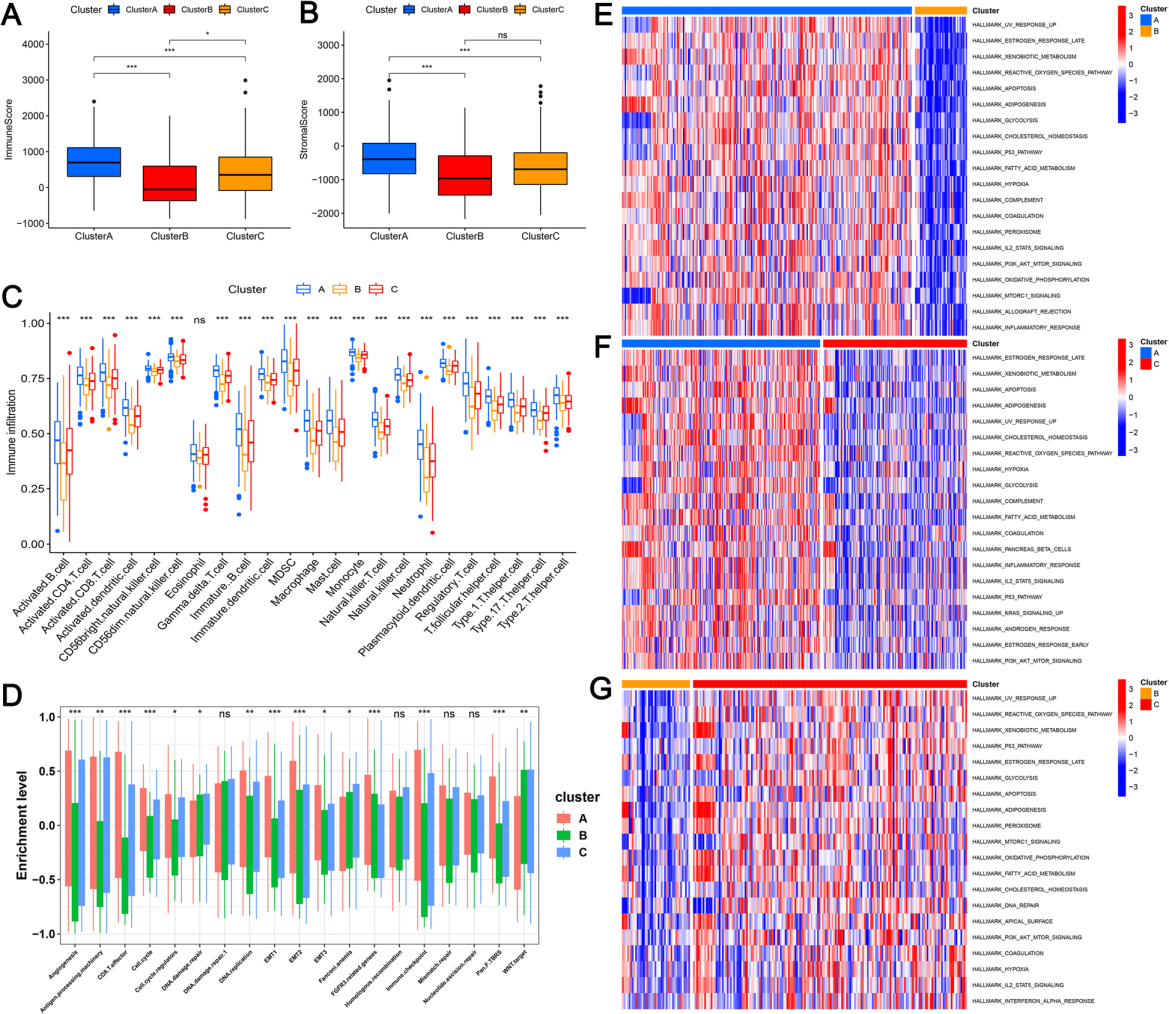
Differences in immune scores and immune cell infiltration between different subgroups. The effect of m6A methylation-related prognostic lncRNAs on the tumor immune microenvironment in patients with COAD was evaluated by assessing the immunization scores, stromal scores, and immune cell infiltration levels of these lncRNAs with high, low, and medium expression in clusters A, B, and C, respectively (Fig. 3a–c). **a** Immunoscore and **b** stromal score of clusters A–C. **c** Infiltration levels of 23 immune cell types in cluster subgroups. **d** Clusters A–C distinguished by different signatures. **e**–**g** GSVA enrichment showing the activation status of biological pathways under different m^6^A modification patterns, **e** cluster A vs cluster B, **f** cluster A vs cluster C, and **g** cluster B vs cluster C. Heat maps showing these biological processes, with red and blue representing activated and inhibited pathways, respectively. COAD cohort was used for sample annotation. *P < 0.05, **P < 0.01, ***P < 0.001. COAD, colon adenocarcinoma; GSVA, gene set variation analysis; m^6^A: N6-methyladenosine.The results revealed that cluster A was significantly enriched in immune activation-associated pathways, including complement response, inflammatory response, allogeneic transplant rejection and cancer suppressor pathways. Cluster C was similar to A. In contrast, cluster B exhibited inhibition of immune pathways and downregulation of pathways associated with cancer inhibition

To evaluate the effect of m^6^A methylation-related prognostic lncRNAs on the tumor immune microenvironment in patients with COAD, we assessed the immunization scores, stromal scores, and immune cell infiltration levels of these lncRNAs with high, low, and medium expression in clusters A, B, and C, respectively (Fig. 3a–c).

To investigate the molecular regulatory mechanisms underlying the differences in the grouping of two different m^6^A-associated prognostic lncRNAs, we conducted gene set variation analysis (GSVA) enrichment analysis of the hallmark gene set (Fig. 3e–g). The results revealed that cluster A was significantly enriched in immune activation-associated pathways, including complement response, inflammatory response, and allogeneic transplant rejection. Cluster A was also enriched in cancer suppressor pathways such as P53 and apoptosis and in the PI3K/AKT/mTOR, KRAS, IL2/STAT5, and MTORC1 pathways, which contribute to cancer progression. Cluster C was similar to A, with significant enrichment of pathways related to immune activation and tumor suppressor pathways and pathways related to cancer progression, such as P53 and apoptosis, INFα and IL2/STAT5 pathways, PI3K/AKT/mTOR, IL2/STAT5 signaling, and MTORC1. In contrast, cluster B exhibited inhibition of immune pathways and downregulation of pathways associated with cancer inhibition.

The enrichment of the cluster A subtype related to epithelial-mesenchymal transition and transforming growth factor-β is shown in a bar chart (Fig. 3d). This enrichment suggests significant enhancement in mesenchymal activation.

### Clinical significance of consensus clustering of methylation-related prognostic lncRNAs

**Fig. 4:**
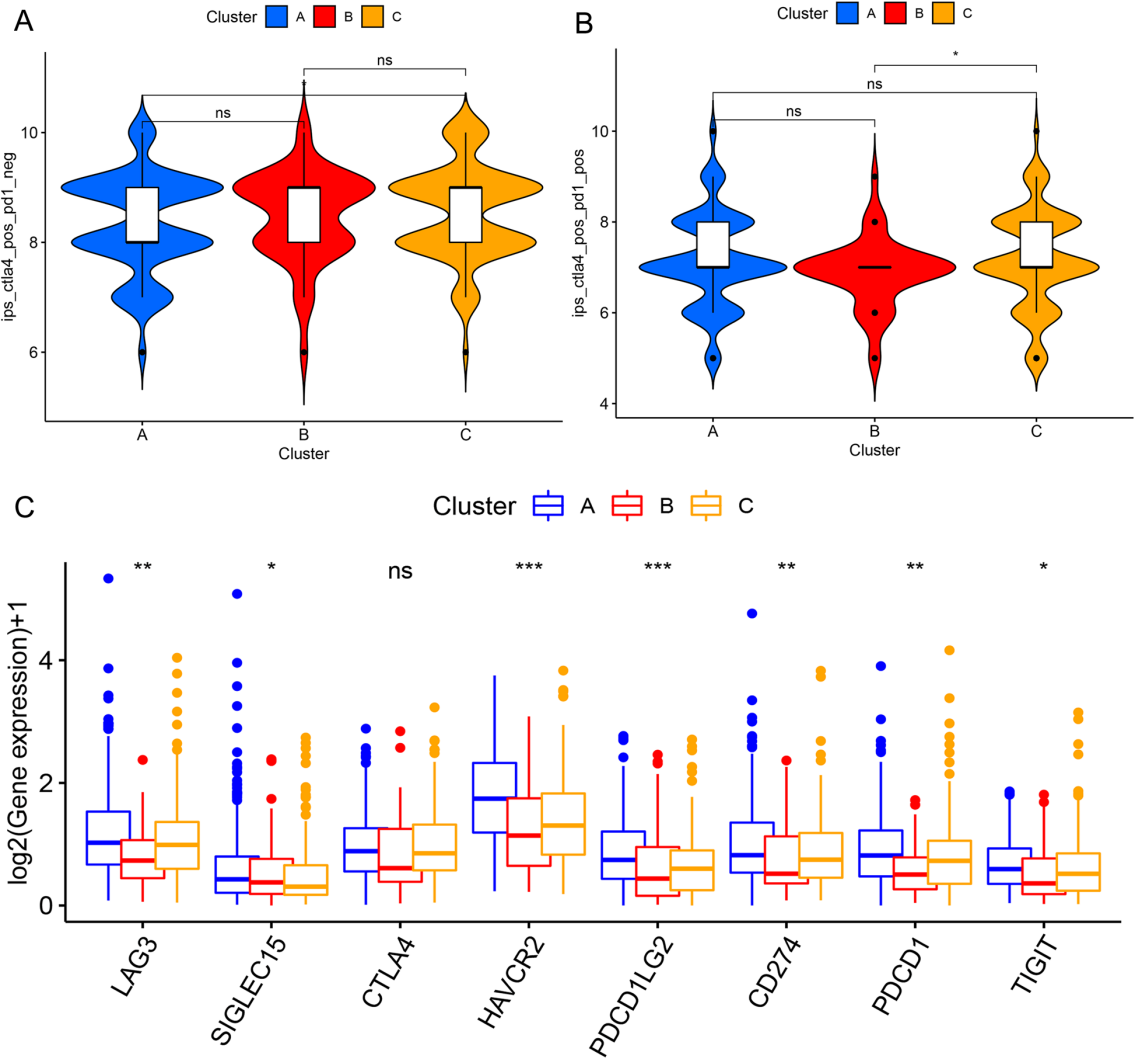
Clusters A–C subtypes correlate with predictors of immunotherapy efficacy. We predicted the difference in immunotherapy between the three clustering subgroups according to the immunophenotypic score (IPS): **a** IPS of *CTLA4* alone effect for clusters A–C subtypes. **b** IPS of clusters A–C subtypes in *CTLA4* and *PD1* combination effect. Cluster C outperformed cluster A when CTLA4 was administered alone (*P* < 0.05), whereas clusters B and C showed no difference in treatment effects. The treatment effect of cluster C was better than that of cluster B following combined therapy with CTLA4 and PD1 (*P* < 0.001); clusters A and C showed no difference in treatment effects. **c** Differential expression of immune checkpoints in clusters A–C. Except for CTLA4 expression, which did not significantly differ among the three subgroups. The expression of all other immune checkpoints differed (HAVCR2, PDCD1LG2: *P* < 0.001; CD274, PDCD1, LAG3: *P* < 0.01; TIGIT, SIGLEC15: *P* < 0.05). The eight immune checkpoints showed the highest expression and immune scores in cluster A. *P < 0.05 and **P < 0.01, ***P < 0.001. *P < 0.05 and **P < 0.01, ***P < 0.001. IPS, immunophenotype score

To determine the clinical significance of m^6^A methylation-related prognostic lncRNAs, we predicted the difference in immunotherapy’s outcome between the three clustering subgroups according to the immunophenotypic score (IPS) (Fig. 4a, b). Cluster C outperformed cluster A when *CTLA4* was administered alone (*P* < 0.05), whereas clusters B and C showed no difference in treatment effects. The treatment effect of cluster C was better than that of cluster B following combined therapy with *CTLA4* and *PD1* (*P* < 0.001); clusters A and C showed no difference in treatment effects. These results suggest that m^6^A methylation-related prognostic lncRNA factors influence immunotherapy outcomes.

The differences in the eight immune checkpoints between cluster subgroups are shown in (Fig. 4c), except for *CTLA4* expression, which did not significantly differ among the three subgroups. The expression of all other immune checkpoints differed (*HAVCR2*, *PDCD1LG2*: *P* < 0.001; *CD274*, *PDCD1*, *LAG3*: *P* < 0.01; *TIGIT*, *SIGLEC15*: *P* < 0.05). The eight immune checkpoints showed the highest expression and immune scores in cluster A.

### Prognostic model of methylation-related lncRNAs and validation

The prognostic role of m^6^A methylation-related prognostic lncRNAs in patients with COAD was examined. We randomly divided 446 patients into TCGA training cohort (270 cases) and verification cohort (176 cases) according to a ratio of 6:4. There were no significant differences in baseline characteristics, age, sex, and TNM stage between TCGA training and testing cohorts (all *P* > 0.05; Table S1).

**Fig. 5:**
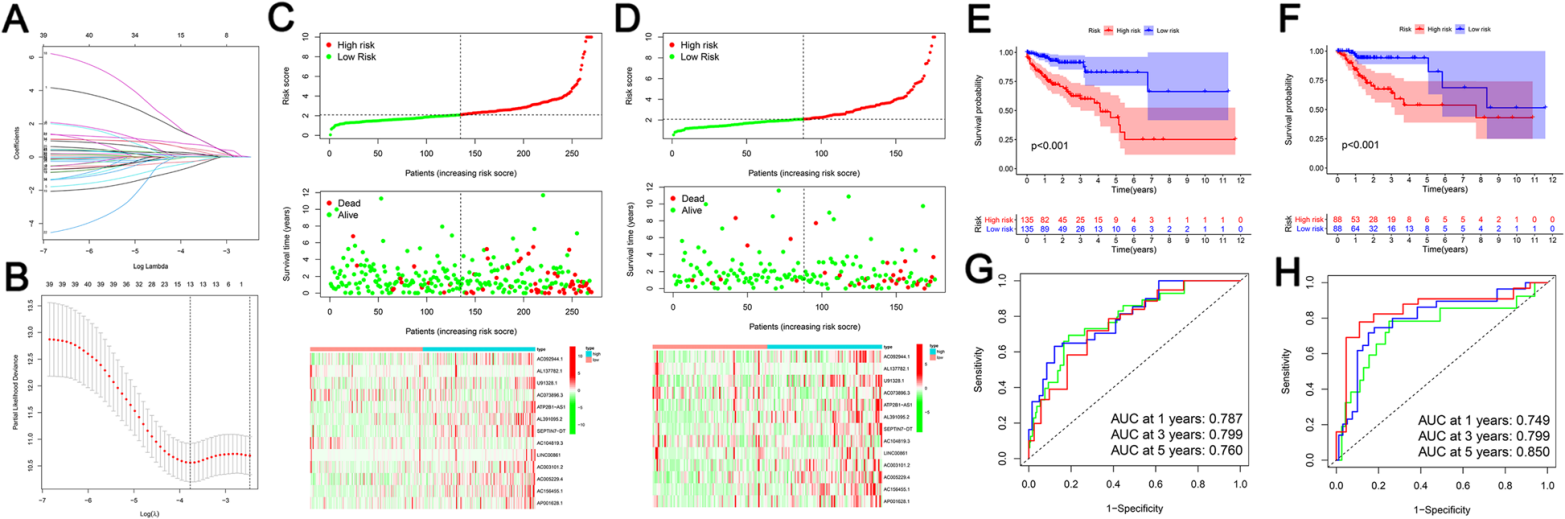
Construction and validation of m^6^A methylation-related prognostic lncRNA prediction signature. **a** and **b** Least absolute shrinkage and selection operator (LASSO) analysis of 43 m6A methylation-associated prognostic lncRNAs in TCGA training cohort was performed, which revealed 13 prognostic predictive signatures: AC092944.1, AL137782.1, U91328.1, AC073896.3, ATP2B1-AS1, AL391095.2, SEPTIN7-DT, AC104819.3, LINC00861, AC003101.2, AC005229.4, AC156455.1, and AP001628.1. The patients were divided into high- and low-risk groups based on the median risk score. **c** and **d** Distribution of risk scores, OS status, and heat maps for 13 predictive m^6^A-associated lncRNAs in TCGA training cohort (**c**) and TCGA validation cohort (**d**). **c**-**d** The f risk scores, OS status, and expression profiles of the 13 prognostic predictive signatures of m6A methylation-associated lncRNAs in the training and validation cohorts. According to the heat map, the prognostic predictors in the high-risk group were significantly higher than those in the low-risk group, except for AL137782.1, AC104819.3, and AC073896.3, which showed lower expression levels than those in the low-risk group. **e** and **f** Kaplan–Meier curves of OS in patients with COAD according to risk scores in TCGA training cohort (**e**) and validation cohort (**f**). In TCGA training and validation cohorts, OS was longer in the low-risk group than in the high-risk group. **g** and **h** ROC curves for risk scores predicting 1-, 3-, and 5-year survival in TCGA training cohort (**g**) and TCGA validation cohort (**h**). In the training cohort, the 1-, 3-, and 5-year AUCs for the 13 prognostic factors were 0.787, 0.799, and 0.760, respectively (**g**). These values in the validation cohort were 0.749, 0.799, and 0.850, respectively (**h**). COAD, colon adenocarcinoma; LASSO, least absolute shrinkage and selection operator; lncRNA, long non-coding RNA; m^6^A: N6-methyladenosine; OS, overall survival; ROC, receiver operating characteristic; TCGA, The Cancer Genome Atlas

To accurately predict clinical outcomes based on m^6^A methylation-associated prognostic lncRNAs in patients with COAD, we performed least absolute shrinkage and selection operator (LASSO) analysis of 43 m^6^A methylation-associated prognostic lncRNAs in TCGA training cohort, which revealed 13 prognostic predictive signatures: *AC092944.1*, *AL137782.1*, *U91328.1*, *AC073896.3*, *ATP2B1-AS1*, *AL391095.2*, *SEPTIN7-DT*, *AC104819.3*, *LINC00861*, *AC003101.2*, *AC005229.4*, *AC156455.1*, and *AP001628.1*. (Fig. 5a, b). The coefficients obtained from LASSO can be used to calculate the risk scores of the training and validation sets, as risk score = (1.2907 × *AC092944.1* expression level) + (-0.3112 × *AL137782.1* expression level) + (0.1480 × *U91328.1* expression level) + (-0.2706 × *AC073896.3* expression level) + ( 0.7747 × *ATP2B1-AS1* expression level) + (0.5129 × *AL391095.2* expression level) + (1.304 × *SEPTIN7-DT* expression level) + (-0.5918 × *AC104819.3* expression) + (0.2377 × *LINC00861* expression level) + (0.4255 × *AC003101.2* expression level) + (0.1462 × *AC005229.4* expression level) + (0.1406 × *AC156455.1* expression level) + (0.0311 × *AP001628.1* expression level) (Table S3).

We then divided the patients into high- and low-risk groups based on the median risk score. Figure 5c, d shows the risk scores, OS status, and expression profiles of the 13 prognostic predictive signatures of m^6^A methylation-associated lncRNAs in the training and validation cohorts. According to the heat map, the prognostic predictors in the high-risk group were significantly higher than those in the low-risk group, except for *AL137782.1*, *AC104819.3*, and *AC073896.3*, which showed lower expression levels than those in the low-risk group. The three m^6^A-related prognostic lncRNAs positively associated with OS were *AL137782.1*, *AC104819.3*, and *AC073896.3*. In TCGA training and validation cohorts, OS was longer in the low-risk group than in the high-risk group (*P* < 0.001, Fig. 5e, f).

To evaluate the prediction accuracy of the identified eight risk signals, we analyzed the receiver operating characteristic curve by comparing the area under the curve (AUC) values of the training and testing cohorts at 1, 3, and 5 years. In the training cohort, the 1-, 3-, and 5-year AUCs for the 13 prognostic factors were 0.787, 0.799, and 0.760, respectively (Fig. 5g). These values in the validation cohort were 0.749, 0.799, and 0.850, respectively (Fig. 5h). The AUCs revealed that the 13 prognostic predictive characteristics of m^6^A methylation-associated lncRNAs could predict the prognosis of patients with COAD.

### Prognostic risk score of COAD correlates with TNM staging and impact of genetic alterations in predicting immune cell infiltration

**Fig. 6:**
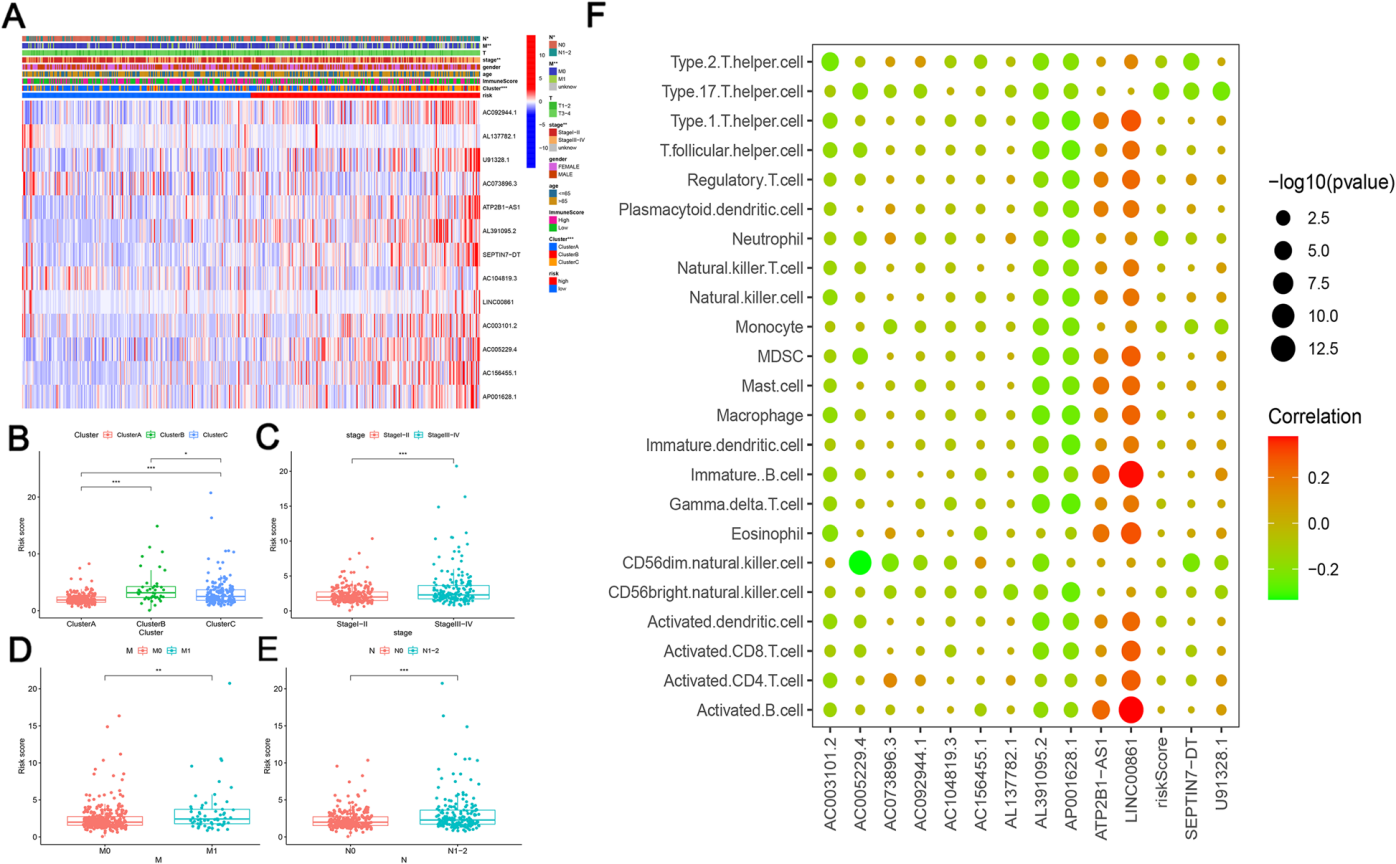
Prognostic risk scores correlated with immune score and TNM staging. **a** Heatmap and clinicopathological features in high- and low-risk groups, except for AL137782.1, AC104819.3, and AC073896.3, which are lncRNAs positively associated with OS, the expression of the other 10 predictors was higher in high-risk group than in the low-risk group. The proportions of cluster subtypes (*P* < 0.001), TNM stage (*P* < 0.01), lymph nodes (*P* < 0.05), and distant metastases (*P* < 0.01) significantly differed between the high- and low-risk groups. (b-c) Distribution of risk scores stratified by **b** clusters A–C, **c** TMN staging, **d** presence of distant tumor metastases, and **e** lymph node metastases. Risk scores were significantly higher in cluster B than in clusters A (*P* < 0.001) and C (**b**). Patients in TNM stages III and VI had significantly higher risk scores compared to those of patients in stages I and II (**c**). Risk scores were higher in patients with distant tumor metastases (**d**) and lymph node metastases (**e**).  **f** Association of methylation-related prognostic lncRNAs predictors and risk scores with immune cell infiltration. The bubble map revealed a relationship between the 13 methylation-related prognostic lncRNA predictors and risk scores and the degree of infiltration of 23 immune cells (**f**). *P < 0.05, **P < 0.01, ***P < 0.001

A heat map was drawn to further evaluate the relationship between the risk score and clinical characteristics. The heat map shows the expression levels of the 13 prognostic predictors in the high- and low-risk groups in TCGA cohort (Fig. 6a). Except for *AL137782.1*, *AC104819.3*, and *AC073896.3*, which are lncRNAs positively associated with OS, the expression of the other 10 predictors was higher in high-risk group than in the low-risk group. The proportions of cluster subtypes (*P* < 0.001), TNM stage (*P* < 0.01), lymph nodes (*P* < 0.05), and distant metastases (*P* < 0.01) significantly differed between the high- and low-risk groups.

We next confirmed the relationship between the risk score, cluster subtype, stage, and other clinical features. Risk scores were significantly higher in cluster B than in clusters A (*P* < 0.001) and clusters C (*P* < 0.05, Fig. 6b), and patients in TNM stages III and VI had significantly higher risk scores compared to those of patients in stages I and II (*P* < 0.001, Fig. 6c). Risk scores were higher in patients with distant tumor metastases (*P* < 0.01, Fig. 6d) and lymph node metastases (*P* < 0.001, Fig. 6e) than in patients with COAD without metastases. These findings suggest that the risk scores of patients with COAD are strongly associated with the disease subtype, TNM stage, lymph nodes, and distant metastases.

The degree of infiltration of 23 immune cell types with risk scores and 13 m^6^A methylation-related prognostic lncRNA predictors was further analyzed to evaluate the effect of risk scores on the tumor immune microenvironment of COAD and explore lncRNA predictors important in immune infiltration. The bubble map revealed a relationship between the 13 methylation-related prognostic lncRNA predictors and risk scores and the degree of infiltration of 23 immune cells (Fig. 6f). *LINC00861* showed a higher and positive correlation with the degree of infiltration of the 23 immune cells.

### Construction and examination of nomograms based on risk scores and clinical attributes

**Fig. 7:**
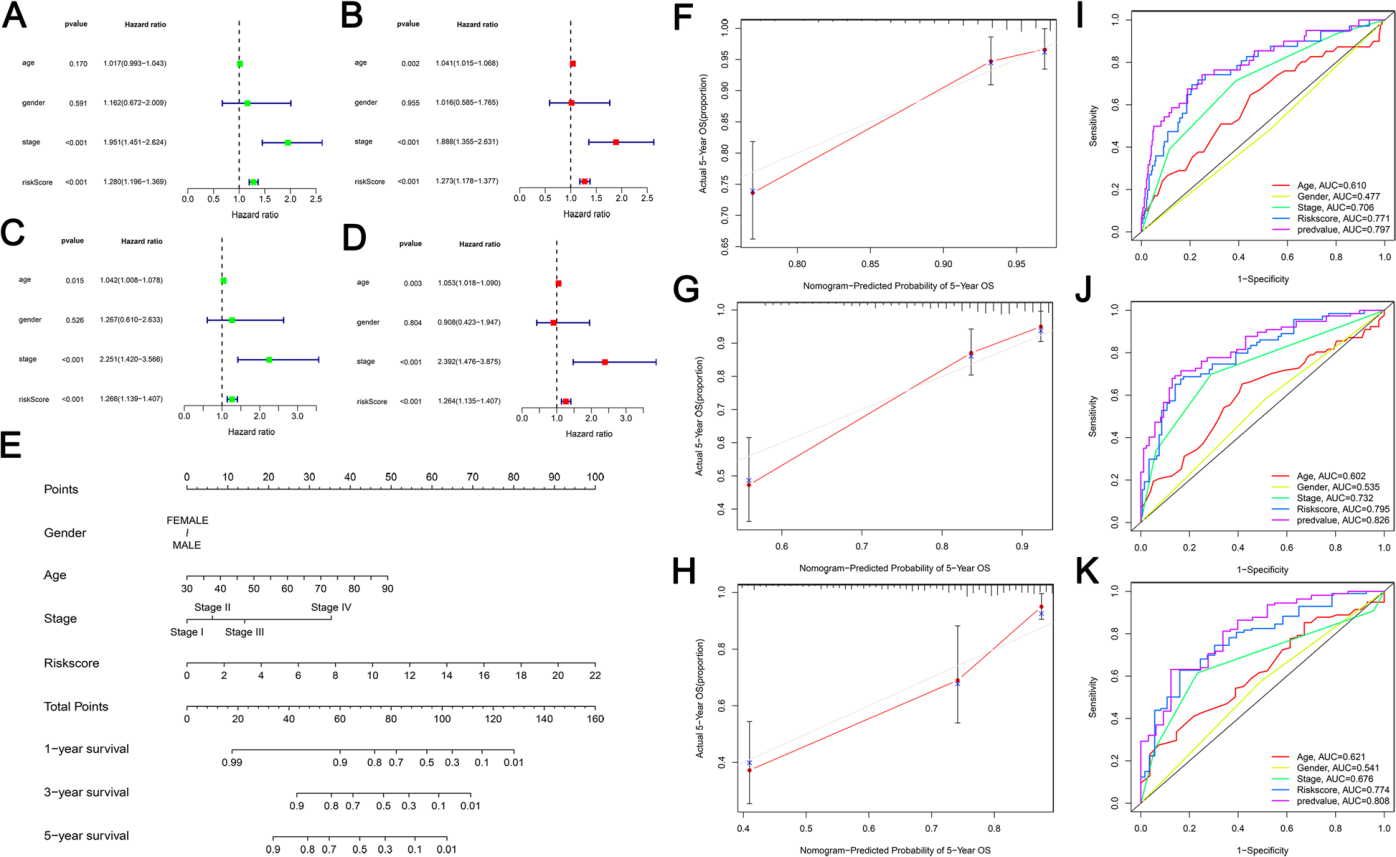
Construction and validation of nomogram. Single-factor and multi-factor Cox regression analyses were performed in the training and validation sets, respectively. In univariate analysis, TNM staging (*P* < 0.001) and risk score (*P* < 0.001) were significantly associated with OS in TCGA training cohort (**a**). TNM stage (*P* < 0.001), risk score (*P* < 0.001) and age (*P* = 0.002) were identified as an independent prognostic factor strongly associated with OS (**b**). In the validation cohort, single-factor Cox analysis showed that the risk score (*P* < 0.001), TNM stage (*P* < 0.001), age (*P* = 0.015), and OS were highly correlated (**c**). TNM stage (*P* < 0.001), risk score (*P* < 0.001), and age (*P* = 0.003) remained significantly associated with OS in single-factor Cox analysis (**d**). A nomogram was constructed that included risk scores and clinical attributes. The risk score, age, sex, and TNM stage were summed to calculate the total score (**e**). The calibration curves for predicting 1-, 3-, and 5-year OS showed that survival predicted by the nomogram was closely related to the actual survival outcome (**f**–**h**). The AUC values at 1, 3, and 5 years were 0.797, 0.826, and 0.808, respectively, which were higher than those for clinical characteristics such as risk score and TNM staging (**i**-**k**)

To determine whether the risk score is an independent prognostic factor in patients with COAD, single-factor and multi-factor Cox regression analyses were performed in the training and validation sets, respectively. In univariate analysis, TNM staging (*P* < 0.001) and risk score (*P* < 0.001) were significantly associated with OS in TCGA training cohort (Fig. 7a). These factors were then included in multifactorial Cox regression analysis, which showed that TNM stage (*P* < 0.001) and risk score (*P* < 0.001) remained strongly associated with OS; interestingly, after excluding confounding factors, age was also identified as an independent prognostic factor strongly associated with OS (*P* = 0.002) (Fig. 7b).

In the validation cohort, single-factor Cox analysis showed that the risk score (*P* < 0.001), TNM stage (*P* < 0.001), age (*P* = 0.015), and OS were highly correlated (Fig. 7c). Similarly, when these factors were included in Cox analysis, TNM stage (*P* < 0.001), risk score (*P* < 0.001), and age (*P* = 0.003) remained significantly associated with OS (Fig. 7d). Based on these results, the risk score obtained from the expression levels of 13 m^6^A methylation-related prognostic lncRNA predictors was an independent prognostic factor for patients with COAD.

Finally, we constructed a nomogram that included risk scores and clinical attributes. The risk score, age, sex, and TNM stage were summed to calculate the total score (Fig. 7e). The calibration curves for predicting 1-, 3-, and 5-year OS showed that survival predicted by the nomogram was closely related to the actual survival outcome (Fig. 7f–h). The AUC values at 1, 3, and 5 years were 0.797, 0.826, and 0.808, respectively, which were higher than those for clinical characteristics such as risk score and TNM staging (Fig. 7i–k).

### Genetic alterations in m^6^A methylation-associated prognostic lncRNAs are associated with predictive markers of immunotherapy efficacy

**Fig. 8:**
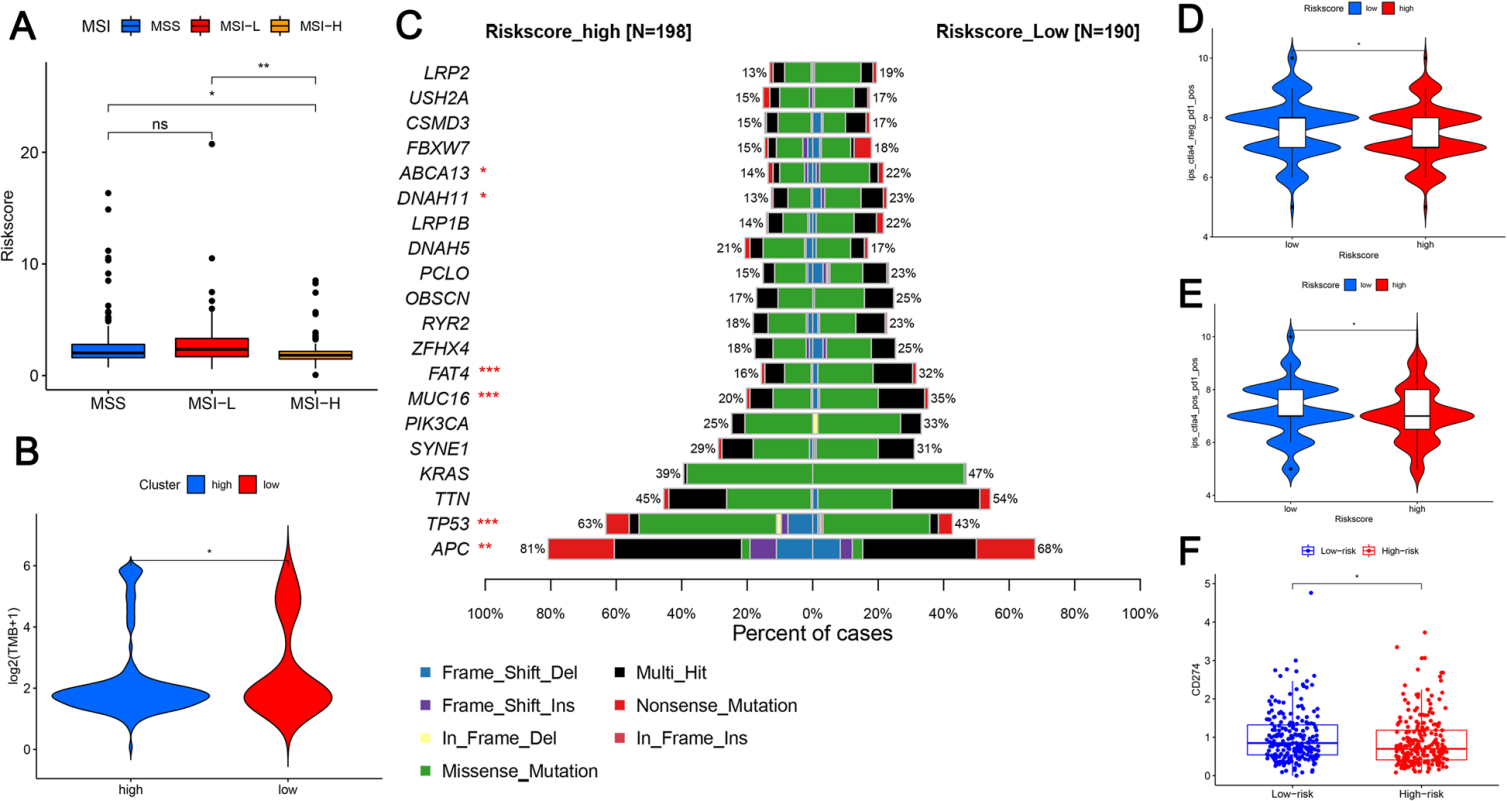
Relationship between risk score and microsatellite instability, tumor mutation load, and immune checkpoints. **a** Microsatellite instability. Patients with MSI-H had lower risk scores, whereas patients with COAD with MSI-L (*P* < 0.01) and MSS (*P* < 0.05) had higher risk scores. **b** Tumor mutational burden.The tumor mutational burden of subgroups with different risk scores was compared and the lower tumor mutation burden in the high-risk score group than in the low score group was observed (*P* < 0.05). **c** We further conducted significantly mutated gene (SMG) analysis of COAD samples from the low-and high-risk scoring subgroups. IPS for **d** *PD1* alone and **e** *PD1* combined with *CTLA4* in the high and low scoring groups. The IPS of PD1 alone or in combination with CTLA4 was higher in the low-risk scoring group than in the high-scoring group (**d**-**e**). **f** Differences in *CD274* expression between the high- and low-risk groups and *CD274* expression was significantly higher in the low-risk group. *P < 0.05 and **P < 0.01, ***P < 0.001. IPS, immunophenotype score; SMG, significantly mutated gene

Patients with MSI-H had lower risk scores, whereas patients with COAD with MSI-L (*P* < 0.01) and MSS (*P* < 0.05) had higher risk scores (Fig. 8a). We compared the tumor mutational burden of subgroups with different risk scores (Fig. 8b) and observed a lower tumor mutation burden in the high-risk score group than in the low score group (*P* < 0.05).

We further conducted significantly mutated gene (SMG) analysis of COAD samples from the low- and high-risk scoring subgroups (Fig. 8c). The SMG mutation profiles showed that in addition to *TP53* (63% vs. 43%, *P* < 0.001) and *APC* (81% vs. 68%, *P* < 0.01), which had higher somatic mutation rates in the high-risk scoring group, *FAT4*, *MUC16* (*P* < 0.001), *DNAH11*, and *ABCA13* (*P* < 0.05) had higher somatic mutation rates in the low-risk score subgroup (Fisher’s exact test).

In addition, the IPS of *PD1* alone or in combination with *CTLA4* was higher in the low-risk scoring group than in the high-scoring group (Fig. 8d, e) (*P* < 0.05). We further explored the differences in immune checkpoint expression in the risk score groups, which showed that *CD274* expression was significantly higher in the low-risk group (Fig. 8f; *P* < 0.05).

### Screening for sensitive drugs targeting high-risk genes in m^6^A methylation-associated lncRNAs prognostic models

Substance sensitivity analysis was performed by Connectivity map drug online database was performed to evaluate genes identified in differential expression analysis of the high-risk scoring group combined with the low-risk scoring group in the prognostic model. We identified 32 sensitive drugs that inhibited high-risk genes in the prognostic model grouping of m^6^A-associated lncRNAs. Among these drugs, piperidolate, quinostatin, and mecamylamine showed the highest degree of negative enrichment, suggesting that they have the highest sensitivity for suppressing high-risk genes in prognostic models (all *P* < 0.05, Table 1).

**Table 1:** Predicted small molecules in connectivity mapping

rank	cmap name	enrichment	p
1	piperidolate	-0.945	0.00028
2	quinostatin	-0.902	0.01927
3	mecamylamine	-0.876	0.00387
4	prenylamine	-0.845	0.00103
5	bezafibrate	-0.835	0.00131
6	chrysin	-0.824	0.01084
7	crotamiton	-0.8	0.0031
8	ebselen	-0.797	0.01719
9	gliclazide	-0.791	0.00386
10	etomidate	-0.789	0.01909
11	CP-645525–01	-0.746	0.03333
12	canavanine	-0.732	0.03992
13	bethanechol	-0.703	0.01593
14	sulconazole	-0.682	0.02234
15	scoulerine	-0.673	0.02572
16	oxybutynin	-0.671	0.0261
17	epitiostanol	-0.67	0.02668
18	levocabastine	-0.657	0.03149
19	halcinonide	-0.65	0.01278
20	parbendazole	-0.648	0.03621
21	phenoxybenzamine	-0.638	0.04144
22	cyclopenthiazide	-0.631	0.04563
23	dosulepin	-0.627	0.04794
24	suloctidil	-0.623	0.04999
25	edrophonium chloride	-0.576	0.04117
26	amprolium	-0.576	0.04133
27	cefazolin	-0.568	0.04558
28	naloxone	-0.564	0.02576
29	cinchocaine	-0.562	0.04962
30	phenformin	-0.559	0.01335
31	metoclopramide	-0.524	0.04728
32	prochlorperazine	-0.511	0.00016

## Discussion

m^6^A is the most enriched internal epigenetic modification in eukaryotic mRNA [14] and is involved in nearly every step of RNA metabolism, including translation, degradation, splicing, export, folding of mRNA [15] and processing of mRNA and ncRNA [9]. We found that alterations in m^6^A levels affect cancer pathogenesis and development by modulating the expression of tumor-associated genes. The effects of lncRNA in tumors is not uniform, with some molecules acting as carcinogens and others as tumor suppressors. Aberrant expression, mutations, and single-nucleotide polymorphisms in lncRNAs are closely associated with tumorigenesis and metastasis [16].

There is increasing evidence that m^6^A modifications interact with lncRNAs as an important link affecting tumor development. For example, Wu et al. found that the m^6^A-inducible lncRNA *RP11* induces propagation of CRC cells by upregulating *Zeb1* [17]. In addition, Ni et al. showed that the lncRNA *GAS5* interacts with *YAP* and triggers its phosphorylation and degradation to inhibit CRC progression, which is negatively regulated by the m^6^A reader *YTHDF3* [18]. However, the overall characteristics of the TME mediated by the interaction of m^6^A with lncRNAs and their impact on therapy and prognosis are unclear. Therefore, identifying the mode of action of different m^6^A-related lncRNAs in the tumor immune microenvironment can improve the understanding of the effect of m^6^A-lncRNA interactions on anti-tumor immune responses and tumor prognostic features of COAD mediated by m^6^A-related lncRNAs, which will facilitate the development of more effective and precise immunotherapeutic strategies.

First, we divided COAD into three types based on consistent clustering analysis of m6A-associated prognostic lncRNAs. Differences in the tumor immune microenvironment were significant between clusters A, B and C. Clusters A and C with good prognoses had significantly higher immune scores compared to cluster B. The significant difference in survival between clusters A and C may be related to their higher immune scores. However, cluster A did not outperform the other two groups in assessing the IPS of immunotherapy, probably because of the complex TME effects. Immune and stromal cells are influenced by tumor cell and TME interactions that can promote tumor development [19].

Infiltration of 23 immune cell types is generally higher in cluster A than in clusters B and C. However, this cell population also includes some immunosuppressive cells, such as regulatory T cells (Tregs) and myeloid-derived suppressor cells (MDSCs), and some expression associated with tumor bodies is higher in cluster A than in clusters B and C. Recent studies have shown that immune cells prefer to remain in the stroma surrounding tumor cell nests in the stroma rather than penetrate the parenchyma of tumor cells, a phenotype known as the immune rejection phenotype [20]. Additionally, multiple immunosuppressive immune cells are commonly found in the TME of CRC. MDSCs are immunosuppressive cells that are similar to queen bees and promote Treg formation [21]. Additionally, MDSCs often become tumor-associated macrophages [22], even shadowing the risk of death in tumor-ridden patients and increasing the risk of checkpoint inhibitor resistance [23, 24]. In addition, many studies showed that Tregs in tumors can inhibit the proliferation of autologous CD4 and CD8 T cells [^+^^+^^25^] and that the frequency of Tregs is negatively correlated with the expression of interferon (IFN)-γ and IL-2 in tumor tissues [26]. Hence, some studies suggested that Tregs are associated with poor prognosis [27].

Hence, we hypothesized that the higher immune score in cluster A, but not the better IPS and OS than in the other two clusters, resulted from the retention of immune cells in the stroma around the tumor cell nests and from the immune tolerance induced by immunosuppressive cells that do not fully exert immunocidal effects.

The GSVA results suggested that cancer-suppressor pathways, including P53 and apoptosis, were downregulated in cluster B. P53 is an important tumor-suppressor encoded by the oncogene *TP53* and is involved in many vital biological processes, including cell cycle arrest, senescence, and apoptosis [28, 29]. Whereas perturbative deletions or mutations of P53 regulate immune recognition, thereby promoting an immunosuppressive environment through mechanisms such as increased suppressive myeloid cells and Tregs [30].This may be an important reason for the lower OS in group B compared to the other two groups. In addition to the P53 pathway, some immune-related pathways are activated in group C but inactivated in group B, such as the IFN-α response. The IFN-α pathway is activated in group B by promoting IL-21, IFN-γ, IL-15 in immune cells [31–33], and other cytokines in immune cells to drive the maturation of dendritic cells [34], which differentiate CD4 T cells into Th1 [^+^^35^] and increase the activation and cytotoxicity of CD8 T cells [^+^^36^]. IFN-α also mediates these immunomodulatory roles in the absence of intermediate cytokine production [34]. The findings from our study are consistent with previous results, suggesting that differences in the IFN-α response were responsible for the differences immune cell infiltration and prolonged OS in clusters A and C. We also observed inconsistent expression of eight common immune checkpoints in COAD tumors and paraneoplastic tissues. This result suggests that the expression of these immune checkpoints differs between cancer and normal tissues and that there are differences in immune checkpoint expression between individual COAD patients. Clarifying these differences and administering different immune checkpoint treatments may provide different benefits to patients. Further analysis of the differences in immune checkpoint expression between different subgroups could facilitate more effective individualized treatment.

Not only that, we assessed the prognostic value of m6A-associated prognostic lncRNAs in COAD patients and derived 13 prognostic risk factors. Among these predictors, some have been previously described, such as ATP2B1-AS1, whose silencing was reported to block the NFKBIA-mediated NF-κB signaling pathway [37]. *LINC00861* has also been identified as a protective factor in ovarian cancer, as a competing endogenous RNA for *miR-513b-5p* in cervical cancer that regulates the PTEN/AKT/mTOR signaling pathway to inhibit cervical cancer cell progression and as being closely associated with *PD1*, *PD-L1*, and *CTLA4* in prostate cancer [38–40]. *AC003101.2* was predicted to play a role in competing endogenous RNA in CRC [41], and *AC156455.1* was reported as a prognostic predictor associated with genomic instability in renal clear cell carcinoma [42]. *AC005229.4* is also an autophagy-related prognostic indicator in hepatocellular carcinoma, bladder cancer, and endometrial cancer [43–45].

Moreover, by linking the risk score and clinical attributes, we created a nomogram and examined its application using data from TCGA. The nomogram could predict OS in patients with COAD, thus facilitating improved prediction of patient survival and clinical decision-making.

Patients with dMMR/MSI early COAD have a higher prognosis and survival compared to patients with MMR proficient/MSI mutations [46, 47], and have a higher density of tumour-infiltrating lymphocytes, with a stronger anti-tumour immune response [48, 49]. There is an overlap between MSI-H/dMMR and tumours with high TMB; however, a large proportion of CRC patients with high TMB do not show defects in the MMR pathway, making TMB a more inclusive biomarker and revealing more patients who may be good candidates for immune checkpoint inhibitor therapy [50]. However, TMB assessments are costly, TMB scoring has not been standardized, and the applicability of the predicted values to MS/MMR proficiency must be further evaluated [51].

Therefore, we further investigated the TMB, MSI, and IPS characteristics of COAD patients in the risk score group. We found that TMB was higher in the low-risk score group and risk scores were lower in COAD patients with MSI-H status. Furthermore, PD1 or PD1 combined with CTLA4 was better for predicting IPS in the low-risk score group. These results are consistent with several previous studies suggesting that these 13 lncRNAs influence the efficacy of immunotherapy and could be used as a surrogate to improve patient stratification and assess the effect of immunotherapy. Furthermore, an assessment of the top 20 most mutated genes in COAD showed an overall higher mutation frequency in the low-risk group, except for tumor suppressor genes such as TP53 and APC, which showed a higher mutation frequency in the high-risk group; these results are consistent with those of GSVA.

As lncRNAs regulated by TME exhibit dynamic changes, they play an important regulatory role in tumorigenesis. For example, the lncRNA MALAT1 adsorbs miR195 and promotes the development of diffuse large B-cell lymphoma and immune evasion. In addition, overexpression of lncRNA UCA1 protects PDL1 expression from miRNA inhibition and promotes immune escape in gastric adenocarcinoma cells [52, 53].

However, the current knowledge on m^6^A-associated prognostic lncRNAs in COAD remains limited. In the present study, the risk scores of 13 prognostic predictors constructed based on m^6^A-related prognostic lncRNAs were inconsistently associated with infiltration of 23 immune cells. *LINC00861* and *ATP2B1-AS1* were positively associated with immune cell infiltration, whereas the other predictors showed negative correlations. This may explain the lack of differences in immune scores between the high- and low-risk groups.

In addition, we screened for sensitive drugs, such as quinostatin, mecamylamine, and piperidolate, that could inhibit high-risk genes in prognostic models of m^6^A-related lncRNAs. The ability of quinostatin to inhibit the PI3K-MTOR pathway is likely important for its anti-tumor role [54]. Mecamylamine inhibits the α7nAChR/NF-ĸB p100/p52 pathway, promotes apoptosis, and disrupts the anti-inflammatory effect in macrophages, potentially influencing the treatment of COAD [55]. Piperidolate is an anticholinergic agent, and previous studies demonstrated that inhibition of cholinergic receptors can inhibit the proliferation of a variety of cancers, suggesting that piperidolate can be applied in cancer treatment [56].

This study had some limitations. Validated was performed using only TCGA dataset. Further independent COAD cohorts should be evaluated to confirm the role of the detected m^6^A-related prognosis-related lncRNAs in prognostic stratification of COAD. Moreover, the role and mechanism of m^6^A-related lncRNAs in the TME and COAD development must be confirmed in vitro and in vivo. Our results provide a foundation for further experimental studies.

## Conclusions

m^6^A-related lncRNAs are significantly associated with the TME and immune responses. Quantitative assessment of m^6^A-associated lncRNAs in individual tumors will enhance the understanding of the TME and immune checkpoint expression profiles. m^6^A-associated lncRNAs were used to construct a prognostic model with potential therapeutic value that can facilitate prognosis and immunotherapy stratification in patients.

## Materials and methods

### Datasets

RNA-seq (FPKM format) transcriptome data were downloaded from the public database The Cancer Genome Atlas (TCGA) data portal (https://portal.gdc.cancer.gov/).

On March 31, 2021, data from 473 COAD specimens and 41 adjoining normal tissues were downloaded. And data without clear TNM staging as well as survival information were excluded. Finally, 446 patients with COAD with appropriate clinicopathological details were included in the follow-up analysis.

The Masked Somatic Mutation data (varscan. Somatic. Maf) files for the nucleic acid-only variant samples were similarly obtained from the TCGA data portal. Immune epistasis scores (IPS) and microsatellite instability scores were obtained from the Cancer Immunome Atlas download (https://tcia.at/home).

### Acquisition of M6A methylation regulators

We annotated the COAD transcript data obtained by TCGA and divided them into two sections: mRNA expression profile and lncRNA expression profile. Based on the published literature [30, 57–59], we collected and identified 24 m6A RNA methylation regulators from the mRNA expression profile of COAD.And 14,086 lncRNAs were isolated from the gene set of the TCGA database with the criteria of non-coding RNAs.

### Screening of M6A-associated prognostic lncRNAs

To screen out the lncRNAs associated with m^6^A RNA methylation regulators, we test the correlation of m^6^A-related lncRNAs in each dataset with Pearson correlation analysis (|Pearson R|> 0.4, *p* < 0.001). As a result, 1612 m^6^A-related lncRNAs were identified.

Then, we screened a total of 43 prognostic lncRNAs associated with m6A by one-way Cox regression analysis with a criterion of *p* < 0.03. In addition, we determined the differential expression of 43 prognostic lncRNAs associated with m6A in tumor tissues and adjacent normal tissues.

### Consensus clustering of M6A-associated lncRNAs after COAD prognosis

Based on 43 M6A-associated prognostic lncRNAs, we classified COAD patients into different subtypes using the “ConsensusClusterPlus” R package. And then principal component analysis (PCA) was performed to verify gene expression patterns between different COAD isoforms, and scatter plots were plotted using the R package “ggplot2”.

### GSVA functional enrichment analysis

We performed GSVA analysis using the R package ‘GSVA’ to investigate the differences in biological processes between different m^6^A modification patterns. Explicit biological features were derived from the Hallmark gene set [47] (downloaded from MSigDB database v7.1) and Mariathasan et al. constructed gene sets [58] (selected from the IMLIGN210CoreBiology package).

### Immune microenvironment and immunotherapy benefit analysis

Estimation of stromal and immune cells in malignant tumors was performed using an expression data (ESTIMATE) algorithm [60], which uses the unique properties of transcriptional profiles to infer tumor cell density and tumor purity. By using the R “ estimate ” package to perform the algorithm, we calculated immune and mesenchymal scores to predict the level of infiltrating immune and stromal cells.

Immunoepitope score (IPS) is a better predictor of response to anti-CTLA-4 and anti-PD-1 options [61]. We determined the differences in their CTLA-4 and anti-PD-1 options by comparing IPS in different clusters and high- and low-risk cohorts.

Relative abounds of 23 immune cell types in the tumor microenvironment were measured using single-sample gene set enrichment analysis (ssGSEA) as a way to compare different levels of immune infiltration between clustered subtypes and risk score subgroups.

### Construction of M6A-related prognostic lncRNA prognostic model

The least absolute shrinkage and selection operator (LASSO) was used to construct the best risk model for methylation-associated lncRNAs. Risk scores were then calculated for each patient based on the model. Patients with COAD cancer were classified into high- and low-risk groups by the median of the risk scores.

### Tumor mutational burden analysis of M6A-associated prognostic lncRNAs

In addition to this, Perl scripts were used to calculate mutation frequency and variant/exon length (38 million) for each sample as a way to compared differences in tumor mutation load between clustered subtypes and risk score groupings [34]. And the “maftools” [35] R package was used to visualize the mutation types.

### Screening for sensitive drug molecules

Differential analysis was performed by limma R package for high and low risk groups, and then risk differentially expressed genes were screened by adjusting for *P*-values less than 0.05 and logFC absolute values greater than 1. Finally, the differentially expressed genes were uploaded to association mapping (https://portals.broadinstitute.org/cmap/) to screen for sensitive drugs that could inhibit the expression of risk genes in the prognostic model.

### Statistical analysis

We performed statistical tests with version R4.0.3. The lncRNAs correlated with m6A regulators were screened using pearson correlation analysis and the correlation of risk scores with the level of immune cell infiltration was examined.

Kruskal–Wallis, Wilcoxon rank-sum test was used for intergroup comparison between two subgroups and more than two subgroups.

Cox regression models were performed for both univariate and multifactorial analyses to identify the independent prognostic value of clinical characteristics and prognostic predictors. According to the findings of multivariate Cox proportional risk analysis, we made nomogram with the R package “rms” with the aim of predicting total mortality at 1, 3, and 5 years. The individual patient’s prognostic risk can be measured by the score relative to each risk element.

AUC was used to evaluate the predictive effectiveness of m6A-related lncRNAs model and Nomogram survival prediction model for OS at 1, 3 and 5 years.

Categorical variables were used to compare the training and validation groups using chi-square tests. In the SMG analysis, fisher’s exact test was used to examine statistical differences in gene mutations between the high-risk and low-risk groups.

The Kaplan–Meier method was used for the survival curves, and the log-rank test was used for comparison between groups.

## Supplementary Information

**Additional file 1: Table S1.** Comparison of baseline characteristics between The Cancer Genome Atlas test and training groups.

**Additional file 2: Table S2.** Differential expression of m6a regulators in normal and tumor tissues in TCGA samples combined with GTEX samples.

**Additional file 3: Table S3.** Table of M6A-related prognostic gene coefficients for model construction.

**Additional file 4: Figure S1.** Differential expression of m6a regulators in normal and tumor tissues in TCGA samples combined with GTEX samples.

## Data Availability

The data can be downloaded at (https://tcia.at/home and https://tcia.at/home), and the codes used in the current study are available from the corresponding author upon reasonable request. And the Supplementary Raw Data about Figs. 2 and 3, GTEX can be downloaded at https://www.jianguoyun.com/p/DQlloFgQ0pnRChiZ7uMEIAA.

## Abbreviations

m^6^A: N6-methyladenosine
TME: Tumor microenvironment
COAD: Colon adenocarcinoma
CRC: Colorectal cancer
LASSO: Least absolute shrinkage and selection operator
MSI: Microsatellite instability
IPS: Immunophenotypic score
*FAT4*: FAT atypical cadherin 4
*MUC16*: Mucin 16
lncRNA: Long non-coding RNA
PD-1: Programmed death receptor 1
PD-L1: Programmed cell death 1 ligand 1
ncRNA: Non-coding RNA
TCGA: The Cancer Genome Atlas
*RBM15B*: RNA-binding motif protein 15B
*ALKBH5*: AlkB homolog 5
*PDCD1LG2*: Programmed cell death 1 ligand 2
*LAG3*: Lymphocyte activating 3
*SIGLEC15*: Sialic acid-binding Ig-like lectin 15
*CTLA4*: Cytotoxic T-lymphocyte associated protein 4
*CD274*: Programmed cell death 1 ligand 1
*TP53*: Tumor protein P53
*APC*: APC regulator of WNT signaling pathway
*DNAH11*: Dynein axonemal heavy chain 11
*ABCA13*: ATP-binding cassette subfamily A member 13
AUC: Area under receiver operating characteristic curve
OS: Over survival
Treg: Regulatory T cell
MDSC: Myeloid-derived suppressor cell
GSVA: Gene set variation analysis
dMMR: Mismatch repair deficiency
