# Supplementary material for: m6A-related lncRNA-based immune infiltration characteristic analysis and prognostic model for colonic adenocarcinoma
Source: Hereditas. 2023 Feb 9;160:6. doi: 10.1186/s41065-023-00267-y (PMC9909974; doi:10.1186/s41065-023-00267-y)
Supplement: Supplementary file 1 — Additional file 1: Table S1. Comparison of baseline characteristics between The Cancer Genome Atlas test and training groups. [file 41065_2023_267_MOESM1_ESM.docx]

**Table S1. Comparison of baseline characteristics between the TCGA test and training groups.**

| Variables |  | test | train | p-value |
| --- | --- | --- | --- | --- |
| T stage | T1~2 | 32 | 54 | 0.7241 |
|  | T3~4 | 144 | 216 |  |
| N stage | N0 | 102 | 164 | 0.6595 |
|  | N1~2 | 74 | 107 |  |
| M stage | M0 | 133 | 197 | 1 |
|  | M1 | 24 | 37 |  |
| TNM stage | I-II | 98 | 154 | 0.7852 |
|  | III-IV | 76 | 111 |  |
| Age | <=65 | 64 | 119 | 0.137 |
|  | >65 | 112 | 152 |  |
| Gender | female | 79 | 133 | 0.4413 |
|  | male | 97 | 13 |  |
