# Supplementary figures and images for: m6A-related lncRNA-based immune infiltration characteristic analysis and prognostic model for colonic adenocarcinoma
Source: Hereditas. 2023 Feb 9;160:6. doi: 10.1186/s41065-023-00267-y (PMC9909974; doi:10.1186/s41065-023-00267-y)

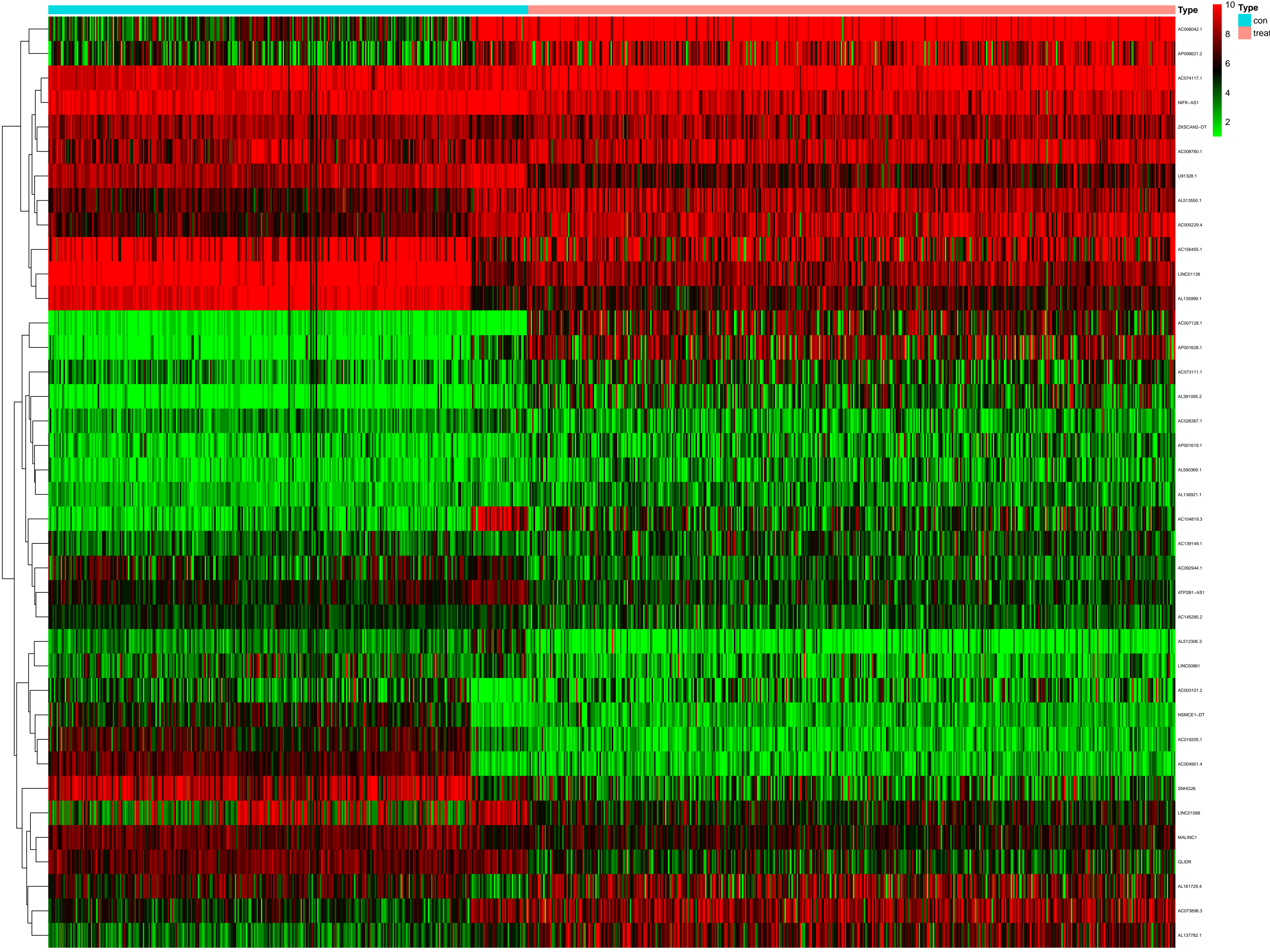

Supplement: Supplementary file 4 — Additional file 4: Figure S1. Differential expression of m6a regulators in normal and tumor tissues in TCGA samples combined with GTEX samples. [file 41065_2023_267_MOESM4_ESM.pdf]
